# The effect of esketamine on sleep disorders in parturients undergoing cesarean section under general anesthesia: a single-center, randomized, double-blind, placebo-controlled prospective study

**DOI:** 10.3389/fmed.2026.1837631

**Published:** 2026-07-31

**Authors:** Hongzhuan Chen, Li An, Pengcheng Zhang, Maoxin Xiao, Qiwei Wang

**Affiliations:** Department of Anesthesiology, Fourth Hospital of Shijiazhuang, Shijiazhuang, China

**Keywords:** cesarean delivery, esketamine, general anesthesia, postpartum depression, postpartum sleep disturbance

## Abstract

**Background:**

This study evaluated the primary effect of esketamine on postoperative sleep disorder (PSD) at 3 days after surgery, as well as its secondary effects on pain and depressive symptoms.

**Methods:**

A total of 140 parturients who underwent elective cesarean delivery under general anesthesia were enrolled and randomized 1:1 to the esketamine or placebo group. After delivery, blinded anaesthesiologists administered a continuous infusion of esketamine (0.3 mg/kg/h) or an equal volume of normal saline. We compared baseline data, anesthesia/surgery indicators, postoperative PSQI scores, sleep NRS scores, incidences of PSD, pain NRS scores and postpartum depression (EPDS) between the groups. We also evaluated adverse reactions and maternal–neonatal complications and performed multivariate logistic regression for PSD risk factors.

**Results:**

At 3 and 7 days after surgery, the PSQI scores, NRS scores, incidence of PSD, and incidence of postpartum depression in the esketamine group were significantly lower than those in the control group (*P* < 0.05). The pain NRS scores at 24, 48, and 72 h postpartum and the rate of rescue analgesia within 72 h were also significantly lower in the esketamine group (*P* < 0.05). Intraoperative consumption of propofol and remifentanil was significantly lower in the esketamine group than in the control group (*P* = 0.038 and *P* < 0.001, respectively). Logistic regression revealed that a 24-h postpartum pain NRS score (OR = 1.485, 95% CI: 1.058–2.084, *P* = 0.022) and antepartum sleep disorder (OR = 4.757, 95% CI: 2.241–10.098, *P* < 0.001) were risk factors for PSD in parturients who underwent cesarean delivery under general anesthesia.

**Conclusion:**

Continuous intraoperative esketamine infusion reduced the incidence of 3-day postoperative sleep disorders and improves sleep quality, while concurrently enhancing analgesic efficacy and ameliorating postpartum depression outcomes in parturients undergoing cesarean section under general anesthesia. Clinicians should prioritize screening for prenatal sleep disturbances and implement early preventive interventions.

**Clinical Trial Registration:**

https://clinicaltrials.gov/, Identifier [ChiCTR23000 78490].

## 1 Introduction

Postoperative sleep disorder (PSD) exhibits a relatively high incidence rate among patients undergoing major surgery, with rates reported in the literature ranging from 30% to 80% (1). This complication, which is primarily characterized by disrupted sleep continuity and insufficient sleep duration, also leads to reductions in the proportions of key sleep stages, such as slow-wave sleep (SWS) and rapid eye movement (REM) sleep.

Cesarean section is a routine obstetric surgical procedure, and clinical data indicate that 30% to 69% of women experience varying degrees of sleep disorders after undergoing cesarean section (2, 3). Postpartum sleep disorders are associated with various factors, such as postpartum pain, newborn crying, breastfeeding, and postpartum anxiety and depression (4–6). Many studies have demonstrated that patients with postpartum depression often exhibit poor sleep quality. Postpartum sleep disorders have multiple adverse effects on mothers, including interfering with the establishment of the mother-infant relationship, causing lactation disorders, and leading to functional impairments at the cognitive level (7). In clinical practice, neuraxial anesthesia is typically administered for cesarean section. However, many parturients demonstrate contraindications to neuraxial anesthesia, thus requiring general anesthesia. Studies have demonstrated that compared with women who receive neuraxial anesthesia, women who receive general anesthesia for cesarean section exhibit a greater risk of developing depression and a greater need for antidepressant medications due to sleep problems (8). Therefore, the early prevention of postpartum sleep disorders and postpartum depression in parturients undergoing general anesthesia is crucial.

At present, many drugs are available for general anesthesia during cesarean section. As the right-handed isomer of ketamine, esketamine not only fully possesses the pharmacological advantages of ketamine but also exhibits a more significant affinity for N-methyl-D-aspartate (NMDA) receptors. Moreover, it can be safely used for general anesthesia in cesarean section surgeries. Studies have demonstrated that esketamine can treat depression and alleviate postoperative pain in surgical patients (9–11); additionally, its rapid antidepressant effect can indirectly improve postpartum sleep disorders (11–15). Moreover, it demonstrates distinct advantages when applied in cesarean section procedures under general anesthesia. However, research investigating the use of esketamine for PSD remains limited. The existing data predominantly originate from small-scale case reports, and there is a significant lack of well-designed randomized controlled trials (RCTs) to verify its efficacy and safety. This dearth of strong evidence has impeded the more extensive clinical application of esketamine in the management of PSD.

This study aimed to evaluate the effect of esketamine on PSD in parturients who underwent cesarean section under general anesthesia on the 3rd postoperative day. The findings present preliminary evidence for the safety and efficacy of perinatal esketamine use, thus providing a foundation for subsequent large-sample, multicentre studies.

## 2 Materials and methods

### 2.1 Study design

**FIGURE 1:**
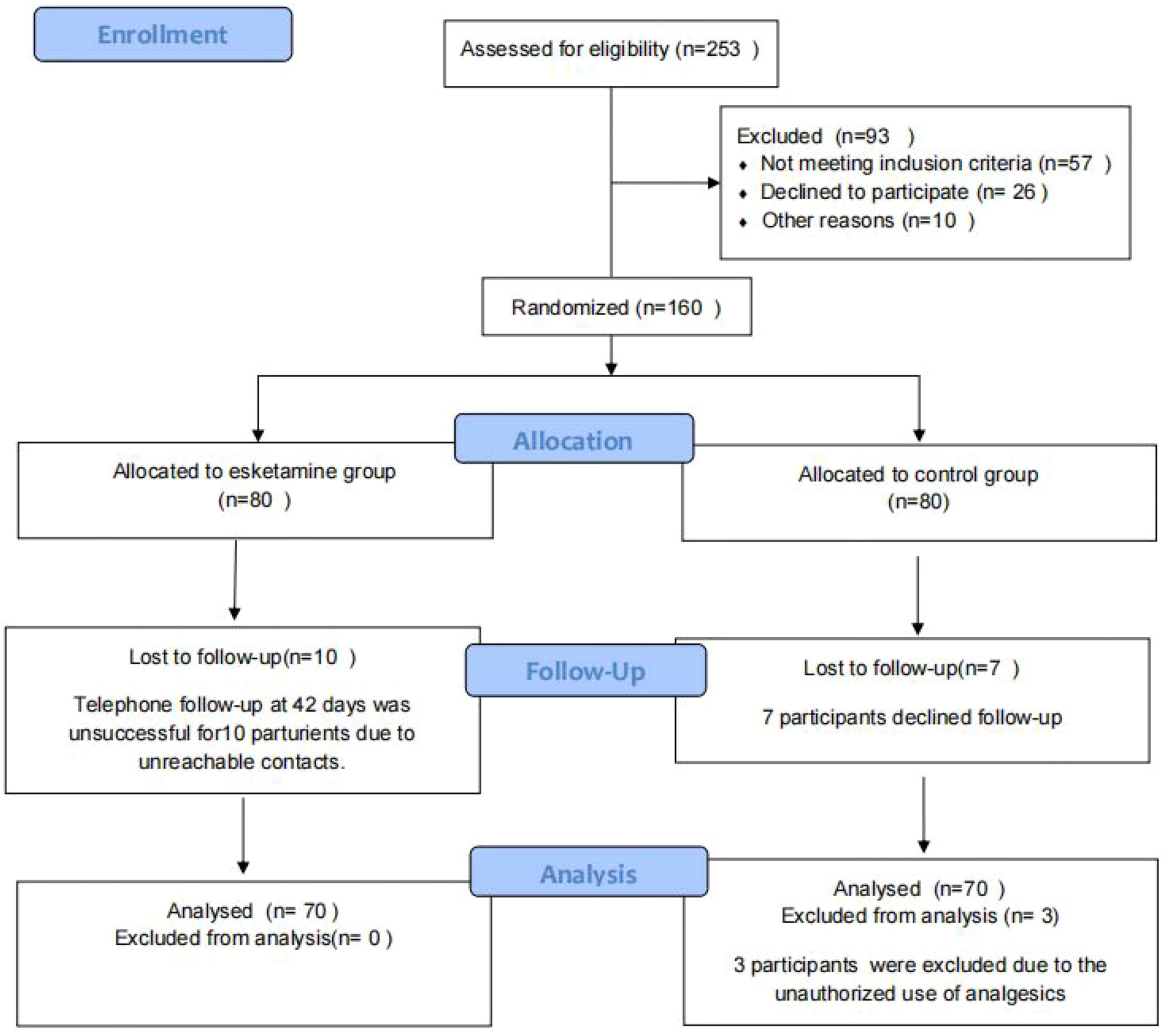
CONSORT 2020 flow diagram.

This randomized controlled trial was approved by the Medical Ethics Committee of Shijiazhuang Fourth Hospital (Ethics Number: 20230170) and registered at the Chinese Clinical Trial Registry (Registration No.: ChiCTR2300078490). This was a single-center, randomized, double-blind, placebo-controlled prospective study conducted at the Fourth Hospital of Shijiazhuang from January 2024 to August 2025. All participants were fully informed of the study procedures and provided written informed consent prior to enrollment. For patients with temporary impairment of decision-making capacity, written informed consent was also obtained from their legally authorized representatives. A comprehensive study flowchart is presented in Figure 1.

### 2.2 Participants

A total of 160 eligible primiparous women were recruited for the study. The following inclusion criteria were utilized: (1) single-term pregnant women (gestational age ≥ 37 weeks) who were scheduled for elective cesarean section under general anesthesia; (2) aged 18–40 years; (3) American Society of Anesthesiologists (ASA) physical status II–III; (4) no history of allergy to esketamine or other anesthetic drugs; and (5) no mental illness or cognitive impairment and able to independently complete the sleep quality assessment. The following exclusion criteria were utilized: (1) a history of allergy to esketamine or other ketamine derivatives; (2) a history of mental diseases (such as schizophrenia, bipolar disorder, or severe depression); (3) severe pregnancy complications (such as preeclampsia, gestational diabetes mellitus with complications, or placental abruption); (4) a history of alcohol or drug abuse within 6 months before pregnancy; (5) a refusal of drug intervention during lactation; (6) a preoperative EPDS score ≥13 points (indicating a severe depressive tendency); (7) other conditions that may affect the study results (such as severe liver and kidney dysfunction or cardiovascular diseases); and (8) a history of stroke, cognitive dysfunction, or epilepsy.

### 2.3 Randomization and blinding

All of the pregnant women scheduled for cesarean section under general anesthesia underwent a qualitative screening 1 day before surgery. The researchers informed the women and their families about the purpose, procedures, benefits, possible risks, and risk-handling methods during the study. A variable block randomization scheme was subsequently adopted to evenly allocate pregnant women who met the inclusion criteria to the esketamine experimental group and the normal saline control group at a 1:1 ratio. A double-blind design was applied to participants, researchers, data collectors, and statisticians. Independent nurses who were not involved in clinical care or data evaluation handled drug dispensing and labeling to ensure allocation concealment. The experimental group received esketamine (Hengrui Pharmaceutical, China) diluted to 1 mg/mL with normal saline in syringes that were identical in appearance to the control group’s normal saline, which were labeled only with the designation “experimental drug + random code”, in order to prevent unblinding based on appearance. Blinding was maintained until completion of all of the final statistical analyses; emergency unblinding was permitted only for severe esketamine-related adverse events (with principal investigator approval) to prioritize participant safety.

### 2.4 Anesthesia and intervention protocol

All of the participants were required to fast for 8 h and abstain from drinking for 2 h before surgery. After the patient entered the operating room, vital signs (blood pressure, heart rate, electrocardiogram, pulse oxygen saturation) received routine monitoring. After the operating surgeon completed skin disinfection and drape placement, general anesthesia induction was initiated; specifically, both groups were administered 1 mg/kg propofol, 4 ng/mL remifentanil target-controlled infusion (TCI) in the effect chamber, and 0.6 mg/kg rocuronium via intravenous injection. A size 3 laryngeal mask airway (LMA) was then inserted, followed by mechanical ventilation. Following fetal delivery, total intravenous anesthesia was intraoperatively maintained with a combination of propofol, remifentanil, and the investigational drug. Anesthesia depth was dynamically adjusted according to bispectral index (BIS) monitoring, with BIS values being maintained between 40 and 60. The investigational drug was infused at a constant rate of 0.3 mL/(kg⋅h) throughout the entire surgical procedure. Postoperatively, ultrasound-guided bilateral transverse fascial plane blocks were administered, with 20 mL of 0.375% ropivacaine injected per side. Once both groups met the extubation criteria, the laryngeal mask was removed. Postoperative analgesia was standardized with patient-controlled intravenous analgesia (PCIA), with the following parameters being implemented: a background infusion rate of 2 mL/h, a bolus dose of 0.5 mL, and a lockout time of 15 min. Rescue analgesia was administered as an intravenous injection of 1 mg of butorphanol tartrate for inadequate postoperative analgesia [numeric rating score (NRS) of pain ≥ 4].

### 2.5 Data collection and outcome assessment

#### 2.5.1 Primary outcomes

The primary endpoint of this study was the incidence of sleep disorders on the third day after surgery. The criteria for diagnosing sleep disorders involved the presence of any one of the following conditions: ① a subjective sleep quality NRS score ≥6 points (16, 17) or ② a total PSQI score >5 points (18, 19). The assessment nodes for subjective sleep quality after surgery were set on the 3rd day, the 7th day, and the 42nd day after surgery. The utilized assessment tools included the NRS and the PSQI. The NRS score ranges from 0 to 10, with 0 corresponding to the best sleep quality and 10 corresponding to the worst sleep quality. The PSQI scale encompasses 18 self-assessment items and 5 other-assessment items. It comprehensively evaluates the subjective sleep status from 7 dimensions, including sleep quality, sleep latency, sleep duration, sleep efficiency, sleep disorders, use of hypnotic drugs, and daytime function. The total score of the scale ranges from 0 to 21, and a higher score indicates worse sleep quality.

#### 2.5.2 Secondary outcomes

The secondary and other endpoint indicators included the following: ① the NRS score, PSQI score, and EPDS score on the third, seventh, and 42nd days after surgery; ② pain being assessed by using the Numeric Rating Scale (NRS) at 24, 48, and 72 h after surgery (pain intensity was rated on an 11-point scale from 0 to 10, where 0 indicated no pain and 10 represented the most severe pain, and parturients were instructed to select a number that best matched their perceived pain level), with the rate of rescue analgesia within 72 h being recorded; ③ the situation of exclusive breastfeeding; and ④ the occurrence of complications within 42 days for both the mother and the infant.

#### 2.5.3 Data collection

Baseline characteristics included age, body mass index, and parity among parturients who underwent cesarean section under general anesthesia. Prenatal assessments were conducted, including the determination of sleep quality in the previous month (assessed using the Pittsburgh Sleep Quality Index [PSQI]); an NRS (11-point scale where 0 = best sleep and 10 = worst sleep) for evaluating subjective sleep quality the night before surgery; and the Edinburgh Depression Scale (EPDS) score for assessing the presence of prenatal depression.

Intrapartum maternal data included the duration of anesthesia, fluid infusion volume, estimated blood loss, duration of surgery, dosage of propofol, dosage of remifentanil, and time to laryngeal mask airway (LMA) removal (defined as the interval from the end of surgery to LMA extubation).

Postoperative complications and adverse reactions were recorded, including adverse events such as nausea and vomiting, dizziness, shivering, diplopia, nightmares, and somnolence, and symptomatic treatment was administered in accordance with clinical diagnosis and treatment guidelines.

### 2.6 Statistical analysis

PASS V.15 software (NCSS, LLC, USA) was used to calculate the sample size based on the primary endpoint. Previous studies have demonstrated that the incidence of postpartum sleep disorders among mothers can reach 69.2% (3). The pre-experimental results of this study suggest that the incidence of sleep disorders in mothers on the third day after delivery is approximately 65%, whereas under the intervention measure of intraoperative infusion of esketamine, this incidence can be reduced to 35%. Based on statistical parameter calculations with α = 0.05 and a test power of 90%, the minimum sample size required for this study was determined to be 128 cases. When considering a potential 20% dropout rate during the study, the final plan was to divide the research subjects into two groups, with 80 parturients being recruited for each group.

Data distribution was assessed via the Shapiro–Wilk test. Continuous variables conforming to a normal distribution were expressed as mean (standard deviation), while non-normally distributed variables were presented as median (interquartile range). Comparisons between groups for normally distributed continuous variables were performed using the two-sample *t*-test, whereas the Mann–Whitney U test was utilized for non-normally distributed continuous variables, reporting the difference in medians and its 95% CI.

Categorical variables were summarized as frequencies (percentages). For primary and secondary binary outcomes, differences between groups were compared using the two-sample *Z*-test, and the absolute risk reduction (ARR) along with its 95% CI was reported. Comparisons of other categorical variables were conducted using the Chi-square test, with Fisher’s exact test applied when the expected frequency was <5. All effect estimates – including Hodges–Lehmann median differences for continuous variables and risk differences (RDs) for binary outcomes – were calculated as the control group value minus the esketamine group value. Accordingly, positive RDs represent absolute risk reductions (ARRs) favoring the esketamine intervention.

In the exploratory analysis, a multivariable logistic regression model was constructed to identify potential risk factors for postpartum sleep disorders. Candidate variables with a *P*-value < 0.10 in univariate analysis were considered for inclusion, while clinically established confounding factors were forced into the model regardless of their univariate statistical significance. A backward stepwise selection procedure minimizing the Akaike Information Criterion (AIC) was applied to derive the final multivariable model. All statistical analyses were performed using SPSS Statistics software (Version 25.0; IBM SPSS Statistics, Chicago, IL, USA). A two-tailed *P*-value < 0.05 was considered statistically significant for all tests. All secondary and subgroup analyses were considered exploratory. Therefore, no correction for multiple testing was applied to the *p*-values for these outcomes. The results for the primary endpoint are considered confirmatory, while all other findings are presented to generate hypotheses for future research.

## 3 Results

### 3.1 Patient characteristics and baseline information

Between January 2024 and August 2025, a total of 253 patients were screened for the study. Of these, 160 patients completed randomization, and 140 parturients were ultimately included in the analysis; these patients were divided into the esketamine group (*n* = 70) and the control group (*n* = 70) (Figure 1). There were no statistically significant differences observed in the baseline data between the two groups of parturients (*P* > 0.05) (Table 1).

**TABLE 1:** Baseline characteristics and intraprocedural variables.

Variables	Esketamine group (*n* = 70)	Control group (*n* = 70)	*P*-value
Age, y	32.4 ± 4.9	32.2 ± 4.8	0.861
BMI, kg/m^2^	28.4 ± 2.9	28.7 ± 3.6	0.534
Years of education, y			0.478
<9	12 (17.1%)	11 (15.7%)	
9–12	5 (7.1%)	2 (2.9%)	
>12	53 (75.7%)	57 (81.4%)	
ASA physical status classification			0.313
II	63 (90.0%)	59 (84.3%)	
III	7 (10.0%)	11 (15.7%)	
Parity			0.536
1	47 (67.1%)	42 (60.0%)	
2	17 (24.3%)	23 (32.9%)	
3	5 (7.1%)	5 (7.1%)	
5	1 (1.4%)	0 (0.0%)	
Antenatal assessment			
PQSI score	5 (4, 8)	5 (3.75, 7)	0.209
NRS score	5 (4, 6)	5 (4, 7)	0.378
EPDS score	8 (6, 9)	8 (6, 9)	0.589
Prevalence of antenatal sleep disorder	35 (50%)	34 (48.6%)	0.866

Data are expressed as the mean ± SD, median (interquartile range), or number of patients (percentage), as appropriate. BMI, body mass index; ASA, American Society of Anesthesiologists; PSQI, Pittsburgh Sleep Quality Index; NRS, numeric rating scale; EPDS, Edinburgh Postnatal Depression Scale.

### 3.2 Intrapartum information

No significant differences were observed in the operative time, anesthesia duration, estimated blood loss, or laryngeal mask removal time between the two groups. The median intraoperative consumption (IQR) of propofol and remifentanil in the esketamine group was significantly lower than that in the control group (350 [350, 380] vs. 350 [350, 400] mg; *P* = 0.038; 0.7 [0.6, 0.9] vs. 0.8 [0.7, 1.0] mg; *P* ≤ 0.001) (Table 2).

**TABLE 2:** Intraoperative data.

Indicator	Esketamine group (*n* = 70)	Control group (*n* = 70)	*P-*value
Anaesthesia duration, min	65 (62, 73.25)	65 (64.75, 70.50)	0.755
Operation time, min	41 (35, 49.25)	41.50 (36.75, 49.25)	0.327
Propofol dosage, mg	350 (350, 380)*	350 (350, 400)	0.038
Remifentanil dosage, mg	0.7 (0.6, 0.9)*	0.8 (0.7, 1.0)	<0.001
Laryngeal mask removal time, min	8 (8, 9)	8 (7.75, 9)	0.107
Blood loss volume, mL	355 (310, 400)	360 (310, 400)	0.923

IQR, interquartile range.

*P < 0.05 compared with the control group.

### 3.3 Primary outcomes

The incidence of PSD in the esketamine group was significantly lower than that in the control group on both the 3rd and 7th postoperative days (day 3: 38.6% vs. 71.4%; ARR 32.9% (95% CI: 16.5% to 46.9%); *P* < 0.001; day 7: 30.0% vs. 57.1%; ARR 27.1% (95% CI: 10.7% to 41.6%); *P* = 0.001) (Table 3).

**TABLE 3:** Postpartum scores, incidence rates and complications at different follow-up time points.

Indicator	Esketamine group (*n* = 70)	Control group (*n* = 70)	Difference between groups (95% CI)	*P-*value
PSQI score				
3 days postpartum	5 (4, 7)*	8 (5.75, 9)	2 (1, 3)	<0.001
7 days postpartum	5 (4, 6)*	5 (5, 8)	0 (0, 1)	0.031
42 days postpartum	5 (4, 6)	5 (4, 8)	0 (0, 1)	0.066
NRS score				
3 days postpartum	5 (4, 6)*	6 (5, 8)	1 (0, 2)	0.001
7 days postpartum	4 (3, 5.25)*	5 (4, 6)	1 (0, 1)	0.002
42 days postpartum	4 (3, 5)	4 (3, 6)	0 (0, 1)	0.356
Incidence of sleep disturbance				
3 days postpartum	27 (38.6%)*	50 (71.4%)	0.329 (0.165–0.469)	<0.001
7 days postpartum	21 (30.0%)*	40 (57.1%)	0.271 (0.107–0.416)	0.001
42 days postpartum	24 (34.3%)	33 (47.1%)	0.129 (−0.033 to 0.281)	0.122
EPDS score				
3 days postpartum	6 (5, 9)*	8 (6, 10)	1 (0, 2)	0.013
7 days postpartum	7 (5, 8.25)*	8 (5, 12)	1 (0, 2)	0.033
42 days postpartum	8 (6, 10)	8 (6.75, 12)	1 (0, 2)	0.189
Incidence of postpartum depression				
3 days postpartum	5 (7.1%)*	14 (20%)	0.129 (0.014–0.244)	0.026
7 days postpartum	5 (7.1%)*	15 (21.4%)	0.143 (0.026–0.260)	0.016
42 days postpartum	11 (15.7%)	14 (20%)	0.043 (−0.086 to 0.170)	0.508
Exclusive breastfeeding				
3 days postpartum	19 (27.1%)	21 (30%)	–	0.708
7 days postpartum	41 (58.6%)	32 (45.7%)	–	0.128
42 days postpartum	41 (58.6%)	32 (45.7%)	–	0.128
Complications at 42 days postpartum				
Parturient	4 (5.7%)	5 (7.1%)	–	0.730
Neonate	7 (10%)	9 (12.9%)	–	0.595

Continuous data: median (IQR), Hodges–Lehmann median difference (95% CI), Mann–Whitney U test. Binary incidence data: *n* (%), Newcombe absolute risk reduction (ARR, 95% CI), two-sample *Z*-test. All differences represent control group minus esketamine group.

*P < 0.05 compared with the control group.

### 3.4 Secondary outcomes

The subjective sleep quality NRS scores in the esketamine group were significantly lower than those in the control group on postoperative days 3 and 7 (day 3: 5 [4, 6] vs. 6 [5, 8], median difference = 1 [95% CI: 0–2], *P* = 0.001; day 7: 4 [3, 5.25] vs. 5 [4, 6], median difference = 1 [95% CI: 0–1], *P* = 0.002). Similarly, the PSQI scores were significantly lower in the esketamine group compared to the control group on postoperative days 3 and 7 (day 3: 5 [4, 7] vs. 8 [5.75, 9], median difference = 2 [95% CI: 1–3], *P* < 0.001; day 7: 5 [4, 6] vs. 5 [5, 8], median difference = 0 [95% CI: 0–1], *P* = 0.031). The median (IQR) EPDS scores at 3 days postpartum (6 [5, 9] vs. 8 [6, 10], median difference = 1 [95% CI: 0–2], *P* = 0.013) and 7 days postpartum (7 [5, 8.25] vs. 8 [5, 12], median difference = 1 [95% CI: 0–2], *P* = 0.033) were significantly lower in the esketamine group than in the control group. The incidence of postpartum depression in the esketamine group was significantly lower than that in the control group on both the 3rd and 7th postoperative days (day 3: 7.1% vs. 20.0%; ARR 12.9% (95% CI: 1.4% to 24.4%); *P* = 0.026; day 7: 7.1% vs. 21.4%; ARR 14.3% (95% CI: 2.6% to 26.0%); *P* = 0.016) (Table 3).

The median (IQR) NRS pain scores during movement at 24 h (3 [2, 3] vs. 3 [3, 3] points, median difference = 0 [95% CI: 0–1]; *P* = 0.006), 48 h (2 [2, 3] vs. 3 [2, 3] points, median difference = 1 [95% CI: 0–1]; *P* < 0.001), and 72 h (3 [2, 3] vs. 3 [2, 3] points, median difference = 0 [95% CI: 0–0]; *P* = 0.011) after surgery were significantly lower in the esketamine group than in the control group. Additionally, the rescue analgesia rate within 72 h was markedly lower in the esketamine group (12.9% vs. 27.1%; ARR 14.3% (95% CI: 1.0% to 27.1%); *P* = 0.035) (Table 4).

**TABLE 4:** NRS pain scores at different postoperative time points and rescue analgesia rates.

Indicator	Esketamine group (*n* = 70)	Control group (*n* = 70)	Difference between groups (95% CI)	*P*-value
24 h postpartum	3 (2, 3)*	3 (3, 3)	0 (0, 1)	0.006
48 h postpartum	2 (2, 3)*	3 (2, 3)	1 (0, 1)	<0.001
72 h postpartum	3 (2, 3)*	3 (2, 3)	0 (0,0)	0.011
Rescue analgesia rate	9 (12.9%)*	19 (27.1%)	0.143 (0.10–0.271)	0.035

Continuous data: median (IQR), Hodges–Lehmann median difference (95% CI), Mann–Whitney U test. Binary data: *n* (%), Newcombe absolute risk reduction (ARR, 95% CI), two-sample *Z*-test. All differences represent control group minus esketamine group.

*P < 0.05 compared with the control group.

There were no statistically significant differences detected in the incidence of adverse reactions between the two groups of parturients (*P* > 0.05) (Table 5).

**TABLE 5:** Adverse reactions.

Variable	Esketamine group (*n* = 70)	Control group (*n* = 70)	*P*-value
Nausea and vomiting	7 (10%)	6 (8.6%)	0.771
Dizziness	7 (10%)	5 (7.1%)	0.546
Nightmares	1 (1.4%)	0	0.316
Diplopia	2 (2.9%)	0	0.154
Shivering	4 (5.7%)	4 (5.7%)	–
Somnolence	5 (7.1%)	4 (5.7%)	0.730

Data are presented as *n* (%).

### 3.5 Exploratory analyses

**FIGURE 2:**
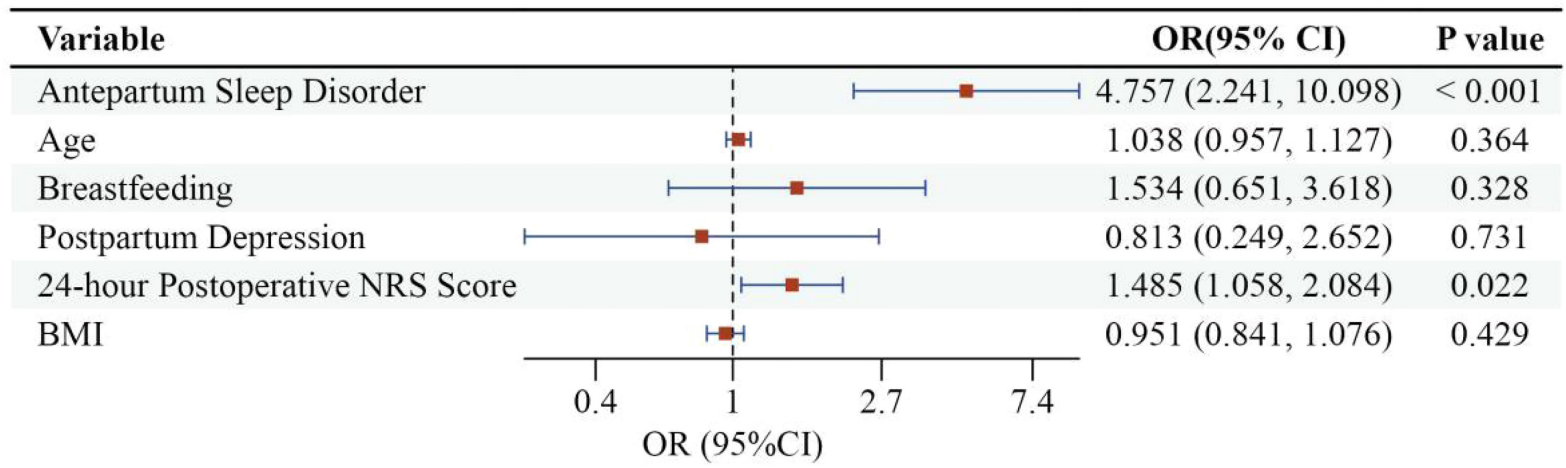
Forest plot of factors analysed for association with incidence of PSD in multivariable logistic regression.

The results of the multivariable logistic regression analysis (Figure 2) revealed that antepartum sleep disorder was an independent risk factor for the study outcome (adjusted OR = 4.757, 95% CI: 2.241–10.098, *P* < 0.001). Moreover, a higher 24-h NRS pain score was significantly associated with an increased risk of the outcome (adjusted OR = 1.485, 95% CI: 1.058–2.084, *P* = 0.022). Conversely, no statistically significant associations were observed between the study outcome and age (adjusted OR = 1.038, 95% CI: 0.957–1.127, *P* = 0.364), breastfeeding status (adjusted OR = 1.534, 95% CI: 0.651–3.618, *P* = 0.328), postpartum depression (adjusted OR = 0.813, 95% CI: 0.249–2.652, *P* = 0.731), or body mass index (BMI; adjusted OR = 0.951, 95% CI: 0.841–1.076, *P* = 0.429).

## 4 Discussion

This study aimed to investigate the effect of esketamine administration during general anesthesia for cesarean section on postpartum sleep quality in parturients. The findings of this study indicated that for parturients who underwent cesarean section under general anesthesia, intraoperative esketamine administration not only reduced the incidence of sleep disorders on the third day postpartum but also improved subjective sleep quality on the third and seventh days postpartum, with a decreasing trend in the incidence of sleep disorders being observed during the same time period. The absolute risk reduction for postoperative sleep disturbance on postpartum day 3 was 32.9% (95% CI: 16.5%–46.9%), indicating that approximately one in every three patients would benefit from esketamine intervention for early sleep impairment. However, this protective effect was not sustained, and no significant intergroup difference in the incidence of sleep disturbance was detected at the 42-day postpartum follow-up. The potential mechanisms underlying these observed effects may involve optimization of postoperative analgesic efficacy and a reduction in the incidence of postpartum depression, although our study was not designed to formally test causal mediation pathways. Postpartum sleep disorders may have long-term effects on both the mother and the baby and are associated with multiple factors. Subsequent related studies should extend the follow-up period to the long-term postpartum stage to further evaluate the sustainability of the benefits and potential risks of this intervention plan.

The results of this study indicated that the NRS scores of pain in the esketamine group on the third day postpartum were significantly lower than those in the control group. Additionally, the rate of rescue analgesia within 3 days after surgery was significantly lower in the esketamine group than in the control group. Moreover, an increase in the NRS pain score at 24 h after surgery was significantly associated with an increased risk of postoperative sleep disorders (OR = 1.485, 95% CI: 1.058–2.084, *P* = 0.022). This result highlights the crucial role of efficient postoperative pain management in improving sleep quality among mothers who undergo cesarean delivery. Previous research has demonstrated that the intraoperative administration of esketamine can significantly reduce postsurgical pain, considerably increase patient satisfaction, and is associated with a lower incidence of adverse effects (20–22). Studies have indicated that the primary cause of postoperative sleep disturbance (PSD) in surgical patients is pain, which is characterized by prolonged sleep latency and reduced total sleep time (23). In contrast, women who undergo cesarean section under general anesthesia often experience more severe and prolonged postpartum pain (24). In the early postoperative period of cesarean section, multiple pain stimuli (such as uterine contraction pain and visceral traction pain) can activate the sympathetic nervous system of the body, thus leading to elevated levels of stress hormones, including plasma epinephrine and cortisol levels. This effect directly disrupts the normal function of the sleep center, thereby prolonging sleep latency and shortening deep sleep duration; conversely, it increases the frequency of nocturnal awakenings in parturients and disrupts sleep continuity (25). Therefore, special attention should be given to postoperative pain and postpartum sleep quality in parturients who undergo cesarean section under general anesthesia. Esketamine can exert direct analgesic effects by blocking the central conduction of pain signals; moreover, it can downregulate the activity of NMDA receptors, reduce the degree of central sensitization, and inhibit the sensitization effect of the pain transmission pathway induced by anesthetic drugs, thereby effectively increasing the pain threshold of the body and reducing the risk of postoperative hyperalgesia. Moreover, this drug can produce a synergistic analgesic effect with opioid drugs, thereby reducing the clinical dosage of opioids. This effect is consistent with our research results. The consumption of propofol and remifentanil during the operation was lower in the esketamine group than in the control group. Collectively, these findings suggest that the intraoperative administration of esketamine may alleviate visceral and incisional pain, potentially contributing to improved sleep quality in postpartum women; however, formal mediation analysis is required to confirm this pathway.

Pregnancy and peripartum sleep disorders are characterized by a critical bidirectional relationship. Pregnancy induces comprehensive physical, psychological, and endocrine remodeling, while gestational discomforts – including uterine enlargement, increased nocturia, and emotional fluctuations – continuously disrupt normal sleep architecture. Conversely, untreated prenatal sleep disturbance aggravates maternal stress hyperresponsiveness and emotional dysregulation, further exacerbating postoperative sleep dysfunction and establishing a vicious cycle; this is consistent with our finding that prenatal sleep disturbance serves as an independent risk factor for PSD. Furthermore, pregnancy-related hormonal balance exhibits significant time-dependent dynamic changes that govern maternal sleep adaptation. Elevated progesterone exerts sedative effects to maintain sleep stability during pregnancy; however, its sharp postpartum decline, combined with surgical pain-induced hyperactivation of the hypothalamic-pituitary-adrenal (HPA) axis and elevated stress hormones, suppresses central sleep function and aggravates sleep fragmentation. This hormonal imbalance typically resolves within 1–2 weeks postpartum under normal conditions, whereas severe postoperative pain and baseline sleep disorders can significantly delay endocrine recovery and prolong poor sleep quality. Future studies should incorporate serial measurements of relevant hormones (e.g., estradiol, progesterone, cortisol) to directly investigate whether these postpartum hormonal fluctuations modify the efficacy and safety profile of esketamine over time. Importantly, systemic anesthetic treatment plays a decisive role in ameliorating perinatal sleep disturbance, demonstrating superiority over single local symptomatic interventions. As a systemic NMDA receptor antagonist, intraoperative esketamine exerts multi-target regulatory effects: it effectively blocks pain signal transmission, alleviates stress hormonal dysregulation, reduces the consumption of conventional anesthetics to avoid residual central nervous system inhibition, and ameliorates postpartum anxiety and depressive symptoms. This systemic intervention may effectively disrupts the vicious cycle of pain, hormonal imbalance, emotional dysregulation, and sleep disturbance, thereby fundamentally improving postpartum sleep quality in parturients undergoing cesarean section under general anesthesia.

This study further demonstrated that esketamine significantly reduced EPDS scores and the incidence of depression among postpartum women on postoperative days 3 and 7. These findings align with the results of previous clinical studies. Specifically, esketamine exerts favorable effects on relieving postoperative pain, alleviating peripartum anxiety and depression, and improving sleep quality after cesarean section, without increasing the overall incidence of adverse reactions (26). Notably, beyond its direct analgesic effect, esketamine also delivers additional benefits in alleviating postpartum depression (27, 28). Li et al. conducted a randomized double-blind controlled trial and verified that esketamine used as an adjuvant analgesic significantly reduced postoperative movement-evoked pain, lowered 42-day postpartum depression incidence and EPDS scores, and decreased opioid consumption after cesarean delivery (27). Furthermore, a recent systematic review and meta-analysis confirmed that esketamine exerts reliable analgesic effects on both resting and movement-induced pain, and is correlated with decreased depressive symptoms among parturients, with an overall favorable safety profile (28). Collectively, this evidence indicates that adequate analgesia with esketamine yields multiple clinical benefits, including improved maternal-infant bonding, a shorter hospital stay, and a lower risk of PPD. By relieving acute postoperative pain, esketamine helps promote early maternal mobilization and mother-infant interaction, thereby simultaneously targeting several key risk factors for postpartum depression. Esketamine may improve postpartum depressive mood through its analgesic effect, thereby potentially contributing to enhanced sleep quality. Studies have confirmed that the alleviation of postpartum depressive symptoms is associated with increased sleep efficiency among parturients (29). We therefore interpret the reduced incidence of sleep disorders in the esketamine group in this study as being associated with the decreased incidence of postpartum depression (30), rather than inferring a direct causal pathway.

A large number of studies have identified numerous clear risk factors for the occurrence of sleep disorders among postpartum women after cesarean section, including advanced maternal age; prepregnancy emotional and sleep conditions; pregnancy complications; anesthesia and operation duration; degree of postpartum pain; postoperative recovery effect; burden of newborn care; breastfeeding status; environmental stress; and other factors related to physical discomfort (31, 32). In this study, multivariate logistic regression analysis revealed that prenatal sleep disturbance and the 24-h postoperative NRS pain score were independent risk factors for PSD among parturients who underwent cesarean section under general anesthesia. It should be noted that this regression analysis was exploratory in nature; given the limited number of outcome events, the model may be prone to overfitting, and these findings should be validated in larger prospective cohorts. Prenatal sleep disturbance was identified as a strong risk factor for postoperative sleep disturbance (OR = 4.757, 95% CI: 2.241–10.098, *P* < 0.001), and these findings were consistent with the conclusions of previous studies on sleep disturbance in parturients undergoing cesarean section (33). Prenatal sleep disorders are often accompanied by long-term sleep rhythm disorders and autonomic nerve function imbalance. Stress factors such as postoperative pain and breastfeeding after cesarean section further disrupt sleep homeostasis. Conversely, prenatal sleep disorders are often accompanied by anxiety and depression, and negative emotions themselves can also lead to postoperative sleep disorders. Therefore, in clinical practice, high-risk groups for prenatal sleep disorders should be screened, and early intervention through psychological intervention and sleep hygiene guidance should be performed to reduce the incidence of PSD (34).

The postoperative analgesic benefits of esketamine are accompanied by certain safety concerns; for example, previous evidence indicates that esketamine can cause psychotic-like reactions such as restlessness, dizziness, headache, nausea, and vomiting (35). To date, relevant literature on the safety of the use of esketamine in infants and young children with lactating mothers receiving this drug remains scarce. With respect to parturients with prenatal depression, existing studies have demonstrated that no serious adverse events occur in breastfed infants after a single low-dose administration of esketamine during the perioperative period (36). In this study, the exclusive breastfeeding status of lactating parturients was tracked throughout the entire study period, and no discomfort or abnormalities were observed among the breastfed infants during the 42-day postpartum follow-up period. However, the potential effects of esketamine exposure through breast milk on the growth and development of infants and young children, as well as its neurotoxic effects, remain unclear and require further verification.

This study has several limitations. First, it is a single-center study. Second, potential confounding factors such as “postoperative breastfeeding frequency” and “level of family support” were not included, which may have introduced bias into the results. Third, the multivariable logistic regression analysis for identifying risk factors was exploratory; given the limited number of outcome events, these findings should be interpreted cautiously as they may be prone to overfitting. Fourth, the assessment of sleep disorders was limited to subjective scales utilized at a single time point, and data support from objective sleep monitoring (e.g., polysomnography) is lacking. Fifth, a considerable number of secondary endpoints were analyzed at several postoperative time points without adjustment for multiple comparisons, which increases the risk of type I error; therefore, these secondary findings should be considered hypothesis-generating rather than confirmatory. Sixth, this study only evaluated short-term (3-day and 7-day) postoperative sleep outcomes, and long-term follow-up data on sleep quality, mood, cognitive function, or infant outcomes are lacking. Although no adverse events were observed in breastfed infants during the 42-day follow-up, the long-term consequences of perioperative esketamine exposure in the peripartum population – including potential effects on neurodevelopment and growth – remain unknown.

Despite these limitations, our findings may have broader implications for sleep disruption arising from other etiologies. Sleep disruption is a well-documented sequela of various clinical conditions, including traumatic brain injury, with consequences ranging from cognitive decline to neurodegeneration (37). Notably, inadequate pain management has been identified as a factor strongly associated with insomnia following traumatic brain injury (37), a finding that parallels our observation that higher postoperative pain scores were correlated with an increased risk of postpartum sleep disorders (OR = 1.485, 95% CI: 1.058–2.084, *P* = 0.022). However, as the present study was not designed to test causal mediation, these relationships should be interpreted as associations rather than causal pathways. The mechanisms by which esketamine alleviates postoperative sleep disturbances – including the modulation of glutamatergic transmission, anti-inflammatory effects, and regulation of the hypothalamic-pituitary-adrenal axis – may therefore extend to other forms of acute sleep disruption in which pain is a driving factor. However, the generalizability of these findings to sleep disorders of different etiologies remains to be established. Furthermore, while the present study focused on cesarean section performed under general anesthesia, the applicability of esketamine to the broader population of parturients undergoing cesarean delivery under neuraxial anesthesia, as well as to vaginal delivery settings – where the anesthetic context, pain pathways, physiological stress responses, and analgesic regimens differ substantially – cannot be directly inferred from our results. Consequently, future research should investigate whether the beneficial effects observed herein replicate in other populations, including those undergoing cesarean section under neuraxial blockade and those undergoing vaginal delivery. Additionally, further studies should address population-level considerations, such as targeted interventions for high-risk groups (e.g., women with antenatal sleep disorders, prenatal depression, or a history of chronic pain).

In future research, we recommend conducting large-sample, multicentre randomized controlled trials to validate the long-term efficacy of esketamine in PSD. The incorporation of a broader range of confounding factors and the integration of objective sleep monitoring technologies would further strengthen the robustness and reliability of the study’s findings. Moreover, interventional studies could be conducted to explore the effect of the combined approach of “prenatal sleep intervention + postoperative multimodal analgesia” on reducing postoperative sleep disorders. Future trials should also incorporate functional endpoints such as maternal-infant bonding assessments.

## 5 Conclusion

In conclusion, this study demonstrates that continuous intraoperative esketamine infusion effectively reduces the incidence of sleep disorders within 3 days following surgery and improves sleep quality in parturients undergoing cesarean section under general anesthesia (ARR: 32.9%, 95% CI: 16.5%–46.9%). These beneficial effects were observed alongside improved postoperative analgesia and a reduced incidence of postpartum depression; however, the study design did not permit the establishment of causal mediation among these outcomes. Furthermore, in clinical practice, priority should be given to screening pregnant women with prenatal sleep disorders and initiating early interventions to mitigate the risk of postpartum sleep disorders.

## Data Availability

The original contributions presented in this study are included in the article/supplementary material, further inquiries can be directed to the corresponding authors.
